# Association Between Nursing Diagnoses and Mortality in Patients with Cardiac Disease: A Retrospective Cohort Study

**DOI:** 10.3390/clinpract16030049

**Published:** 2026-02-26

**Authors:** Vanessa Castellanos-Arreola, Ana Cristina Castañeda-Márquez, Raúl Fernando Guerrero-Castañeda, Dulce Milagros Razo-Blanco-Hernández, Luís Ricardo Vázquez-García, Juan Carlos Fernando Sánchez-Velázquez, María del Carmen Velázquez-Núñez, María Yazmin Castañeda-Ramírez, José Ángel Hernández-Mariano

**Affiliations:** 1 Department of Quality and Health Education, Ministry of Health, Mexico City 11400, Mexico; 2 Department of Research, Hospital Juarez of Mexico, Mexico City 07760, Mexico; 3 Scientific Research Institute, Juarez University of the State of Durango, Durango 34000, Mexico; 4 Department of Clinical Nursing, University of Guanajuato, Celaya 38140, Mexico; 5 Faculty of Nursing, Meritorious Autonomous University of Puebla, Puebla of Zaragoza 72410, Mexico; 6 Nursing School, Hospital Juarez of Mexico, Mexico City 06090, Mexico; 7 Department of Nursing Research, Hospital Juarez of Mexico, Mexico City 07760, Mexico; 8 Coronary Intensive Care Unit, Hospital Juarez of Mexico, Mexico City 07760, Mexico

**Keywords:** heart diseases, nursing diagnosis, cohort study, coronary intensive care units

## Abstract

**Background/Objectives**: Cardiovascular diseases represent a considerable burden on healthcare systems. In coronary intensive care units (CICU), nursing staff play a key role in the care of critically ill patients. Nursing diagnoses (NDs) based on the NANDA-I (North American Nursing Diagnosis Association-International) taxonomy enable the identification of human responses to various clinical conditions. However, their association with adverse outcomes, such as in-hospital mortality, remains understudied. Therefore, we evaluated the association between NDs and in-hospital mortality in patients with cardiac disease. **Methods**: A retrospective cohort study was conducted in a tertiary care hospital. The paper clinical records of 195 patients admitted to the CICU for at least 48 h between January 2023 and March 2025 were reviewed. The association of interest was assessed using Poisson regression models adjusted for confounding variables. **Results**: Mortality was 24.1%. NDs focusing on cardiac and extracardiac responses, such as fluid volume excess (risk ratio [RR] = 2.67; 95% confidence interval [CI] = 1.23, 5.76), impaired cardiac output (RR = 1.84; 95% CI = 1.50, 2.25), risk of shock (RR = 3.12; 95% CI = 1.91, 5.11), risk for impaired cardiovascular function (RR = 2.01; 95% CI = 1.28, 3.17), and impaired gas exchange (RR = 2.67; 95% CI = 1.64, 4.34) were significant predictors of mortality. In contrast, diagnoses such as anxiety (RR = 0.46; 95% CI = 0.23, 0.91), impaired psychological comfort (RR = 0.31; 95% CI = 0.09, 0.95), and risk of unstable glycemia (RR = 0.46; 95% CI = 0.23, 0.91) were associated with a lower risk of death. **Conclusions**: NDs are independently associated with in-hospital mortality in critically ill patients with cardiac disease and may represent useful clinical markers for risk stratification in intensive care settings.

## 1. Introduction

An estimated 17.9 million deaths per year (approximately 32% of all global deaths) are attributable to cardiovascular diseases (CVDs), making them the leading cause of mortality worldwide. Acute myocardial infarction and stroke contribute to approximately 85% of these deaths [1]. Across Latin America, and particularly in Mexico, CVDs also represent a significant public health burden [2]. In 2024, heart disease was the leading cause of death in Mexico, with approximately 145,000 recorded deaths [3].

Given the high mortality associated with these conditions, specialized acute-phase care is essential. Coronary Intensive Care Units (CICUs) are critical for managing patients with acute cardiac events, such as myocardial infarction, decompensated heart failure, life-threatening arrhythmias, or postoperative complications [4,5]. In this setting, nurses play a pivotal role not only in executing procedures but also in assessing and managing patients’ needs. Their responsibilities include monitoring hemodynamic stability, managing fluid balance, administering medications, and detecting early warning signs [6]. This requires clinical judgment, critical thinking, and evidence-based decision-making [7].

The Nursing Care Process (NCP) provides a structured framework for this practice, enhancing professional autonomy and supporting individualized care. It includes five interrelated steps: assessment, nursing diagnosis (ND), planning, implementation, and evaluation [7,8].

Nursing diagnoses (NDs) are clinical judgments about actual or potential responses to health problems that integrate physical, emotional, social, and spiritual dimensions [8,9]. Nursing staff have adopted standardized language systems to unify the terminology used in patient care, facilitating comparison and evaluation of the effectiveness of interventions. Although other recognized taxonomies exist [10,11], NANDA-I (North American Nursing Diagnosis Association-International) is the most widely used classification worldwide [12], particularly in Latin America, where it has been adopted as a reference in numerous academic programs and clinical settings [13,14,15]. In Mexico, institutional efforts since 2007 have promoted the adoption of NDs through educational reforms and national strategies. However, the routine clinical use of NDs remains limited [16,17].

NDs capture individuals’ physical, psychological, social, and spiritual responses to health and illness [8,9]. These responses, identified through comprehensive, continuous assessment, may reflect clinical deterioration and are associated with increased risk of complications, organ failure, or death [18]. Unlike isolated biomedical indicators, NDs synthesize complex patterns of physiological instability and functional decline, allowing them to function as early clinical alerts and to inform predictive models of in-hospital mortality [19].

Several studies have suggested that NDs may predict adverse outcomes such as mortality or prolonged hospitalization, particularly in patients with chronic conditions, postoperative status, cancer, or COVID-19 [18,20,21,22,23,24,25,26,27,28]. However, evidence in cardiovascular populations remains limited, consisting mostly of case reports [29,30,31,32,33,34] or descriptive studies on diagnostic frequency [26,35,36,37,38,39]

Among the limited studies evaluating the association between NANDA-I NDs and mortality, most have included heterogeneous hospital or intensive care populations [23,26,27]. Such variability in case mix may obscure patterns specific to patients with acute cardiac disease, whose pathophysiological profile and risk dynamics differ from those of non-cardiac conditions. Furthermore, most studies have assessed the total number of NDs as a risk factor, without considering the individual contributions of each ND, despite differences in their clinical meanings and potential associations with outcomes [24,25,28]. In this context, identifying NDs associated with increased mortality risk in patients with heart disease may inform early clinical assessment and decision-making by nurses and the broader healthcare team. Generating evidence on the association between individual NDs and in-hospital mortality may also support their more systematic integration into clinical documentation and care planning in Mexico. Therefore, we aimed to evaluate the association between NDs and in-hospital mortality in patients with cardiac disease.

## 2. Materials and Methods

### 2.1. Study Design and Context

This retrospective cohort study was performed at a tertiary referral hospital within the Mexico City Ministry of Health system. The institution offers publicly funded specialized care, mainly to uninsured patients from Mexico City and the State of Mexico. The hospital has a total of 433 inpatient beds, including four allocated to the CICU.

Given the retrospective design and in accordance with reporting recommendations for observational studies [40], all eligible patients admitted to the CICU during the predefined study period were included. Therefore, no a priori sample size calculation was performed, as the analytic sample comprises the entire accessible population (census sampling) within the study timeframe. As described in epidemiologic methodology for secondary data–based cohort studies [41], when the study population is defined by available records, the sample size is determined by data availability rather than by prospective estimation.

### 2.2. Participants

Adults aged 18 years or older, excluding pregnant individuals, who remained hospitalized in the CICU for ≥48 h between January 2023 and March 2025 were considered eligible. All medical records meeting these criteria during the predefined study period were reviewed.

### 2.3. Study Variables and Data Collection

NDs were the independent variables and were coded dichotomously (present/absent) if documented at least once during hospitalization. The frequency of each ND was calculated as the proportion of patients with the diagnosis among the total study population. To ensure appropriate temporal sequencing, only NDs recorded prior to the occurrence of the outcome were included in the analysis. For patients who died, NDs documented after the death event were excluded. This approach minimized the risk of reverse causation or interpretative bias.

The covariates included sociodemographic characteristics (age, sex, education, occupation, and marital status); medical diagnosis and comorbidities; clinical parameters at admission (heart rate, blood pressure, respiratory rate, oxygen saturation, body temperature, and shock index); and length of hospital stay. For descriptive purposes, we grouped medical diagnoses into five categories based on the cardiac condition: ischemic heart disease, rhythm and conduction disorders, valvular heart disease, heart failure and its complications, and postoperative cardiovascular status.

Data were retrospectively extracted from paper-based clinical records, as the hospital does not have an electronic medical record system. Authorization to review the records of patients with heart disease admitted to the CICU during the study period was granted by the Medical Records Department. Eligible records were manually reviewed and digitized into a database developed for this study.

To address potential concerns about interobserver consistency, we conducted an independent validation exercise before applying the exclusion criteria. For this purpose, we extracted and reviewed 15% of the medical records (n = 30). Agreement was assessed using Cohen’s kappa coefficient, which yielded a value of 0.741, consistent with substantial interobserver agreement.

### 2.4. Statistical Analysis

Patient characteristics and NDs were summarized using frequencies and percentages for categorical variables. For continuous variables, normality was assessed with the Shapiro–Wilk test; as distributions were non-normal, medians and interquartile ranges were reported. Comparisons between survivors and non-survivors were performed using Pearson’s chi-squared test or Fisher’s exact test for categorical variables and the Mann–Whitney U test for continuous variables.

Additionally, to evaluate the robustness of the associations and to explore whether the relationship between NDs and mortality varied by clinical severity, we performed a sensitivity analysis assessing effect modification by the shock index [42,43]. The shock index (heart rate/systolic blood pressure), was selected because it provides a rapid, reproducible, and validated measure of circulatory compromise, integrating both cardiac output and vascular tone, and has shown superior predictive performance compared with heart rate or blood pressure alone in identifying critically ill patients at higher risk of adverse outcomes in cardiovascular and intensive care settings [44,45]. For this purpose, an interaction term (shock index × specific ND) was included in the adjusted Poisson regression models, and its statistical significance was evaluated using Wald tests on the multiplicative scale. This approach allowed us to determine whether the association between each nursing diagnosis and mortality differed across strata of physiological severity.

Associations between each ND and in-hospital mortality were assessed using Poisson regression models with robust variance estimation, yielding relative risks (RRs) comparing patients with and without each diagnosis. All models were adjusted for confounders identified through Directed Acyclic Graphs (DAGs) [46,47]. The final adjustment set considered sufficient for confounding control included sex, age, education level, household income, comorbidities, and medical diagnosis (see Supplementary Figure S1). Statistical significance was defined as *p* < 0.05. All analyses were conducted using STATA version 19.5 (StataCorp, College Station, TX, USA).

## 3. Results

A total of 277 medical records were screened for eligibility. After applying the inclusion criteria, 195 patients constituted the final analytic sample. The in-hospital mortality rate was 24.1% (95% CI, 18.5–30.6%). Most participants were men (57.4%) with a median age of 53 years. Compared with survivors, deceased patients were older, more frequently retired, and had lower educational attainment (Table 1).

Ischemic heart disease and heart failure with complications were the leading causes of admission. On admission, the median systolic and diastolic blood pressures were 124 and 70 mmHg, respectively; the median heart rate was 84 bpm, and the respiratory rate was 19 bpm. A shock index > 0.7 was present in 32.8%, and 67.2% had at least one comorbidity. The median hospital stay was 3 days. Non-survivors had significantly higher heart and respiratory rates, shock index, and number of comorbidities, while their oxygen saturation and systolic blood pressure were lower (Table 2).

Nineteen different NDs were identified during hospitalization. The most frequent included risk for decreased cardiac tissue perfusion (81.0%), decreased cardiac output (56.9%), risk for impaired cardiovascular function (33.8%), impaired gas exchange (31.3%), and risk for shock (29.2%; Table 3).

Among non-survivors, a higher proportion had NDs such as risk for electrolyte imbalance, excess fluid volume, impaired gas exchange, decreased cardiac output, risk for ineffective cerebral tissue perfusion, risk for impaired cardiovascular function, risk for shock, and decreased body temperature. In contrast, anxiety, acute pain, impaired psychological comfort, and risk for unstable blood glucose were more frequent among survivors (Supplementary Table S1).

**Figure 1:**
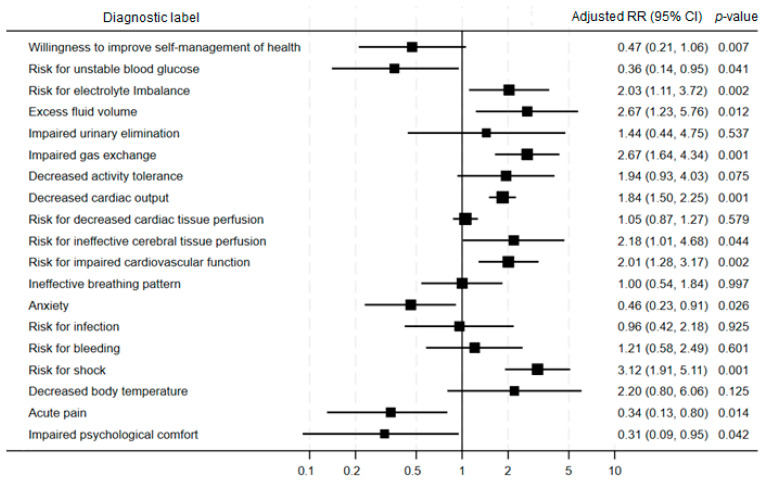
Forest plot for adjusted risk ratios of the association between nursing diagnoses and mortality in patients with cardiac disease. Footnote: Abbreviations: RR, risk ratio; CI, confidence interval. The x-axis displays risk ratios ranging from 0.1 to 10, with RR = 1 indicating no association. Values < 1 indicate an inverse association with in-hospital mortality, whereas values > 1 indicate a positive association.

After adjustment for confounding variables, we found that patients with NDs focused on direct cardiac human responses, such as excess fluid volume (RR = 2.67; *p*-value = 0.012), decreased cardiac output (RR = 1.84; *p*-value = 0.001), risk for impaired cardiovascular function (RR = 2.01; *p*-value = 0.002), and risk for shock (RR = 3.12; *p*-value = 0.001) had a significantly higher risk of mortality. Furthermore, NDs for indirect cardiac and extracardiac responses, such as risk for electrolyte imbalance (RR = 2.03; *p*-value = 0.002), impaired gas exchange (RR = 2.67; *p*-value = 0.001), and risk for ineffective cerebral tissue perfusion (RR = 2.18; *p*-value = 0.044), were also significant predictors of in-hospital mortality. In contrast, NDs such as anxiety (RR = 0.46; *p*-value = 0.026), impaired psychological comfort (RR = 0.31; *p*-value = 0.042), acute pain (RR = 0.34; *p*-value = 0.014), and risk for unstable blood glucose (RR = 0.36; *p*-value = 0.041) were associated with a lower risk of death (Figure 1). Similar results were observed in the unadjusted Poisson regression models (Supplementary Figure S2).

In the stratified analysis, all interaction terms between NDs and the shock index category (<0.7 vs. ≥0.7) were statistically significant (*p* for interaction ≤ 0.005 for all comparisons), indicating that clinical severity modified the association between NDs and in-hospital mortality. For physiological and hemodynamic diagnoses (Risk for electrolyte imbalance, Excess fluid volume, Impaired gas exchange, Decreased cardiac output, Risk for ineffective cerebral tissue perfusion, Risk for impaired cardiovascular function, and Risk for shock), the relative risks were higher among patients with a shock index ≥ 0.7, showing that these NDs identified a subgroup at particularly high risk. Consistently, the magnitude of association of NDs on psychosocial human responses (Anxiety and Impaired psychological comfort) was attenuated among patients with a shock index ≥ 0.7. In contrast, Acute pain, whose association with lower mortality became stronger in the group with higher physiological severity (RR = 0.16; *p* = 0.024; Supplementary Table S2).

## 4. Discussion

In this study conducted on patients with cardiac disease admitted to the CICU, an in-hospital mortality rate of 24.1% was observed. This figure aligns with previous studies on critically ill patients with cardiovascular disease, which report rates ranging from 8.3% to 32.5% [48,49,50]. Despite falling within this range, the observed mortality represents a substantial burden that must be considered in clinical decision-making and the organization of intensive cardiac care. Beyond its magnitude, mortality in this cohort was more frequent among older adults and individuals with low educational attainment, in line with previous research highlighting the critical role of social determinants in shaping adverse hospital outcomes [51,52,53].

Clinically, non-survivors showed greater hemodynamic instability at admission, including higher heart and respiratory rates, a higher shock index, lower oxygen saturation, and lower systolic blood pressure. These alterations reflect more severe functional impairment. Furthermore, the number of comorbidities was significantly higher among non-survivors, highlighting the importance of baseline clinical status as a key determinant of mortality in CICUs [54,55].

In this study, NDs reflecting both cardiac and extracardiac responses were significantly associated with in-hospital mortality, supporting their potential clinical relevance. To our knowledge, this is the first study in Mexico to examine the relationship between individual NDs and in-hospital mortality among patients with cardiac disease. Unlike most prior research, which evaluated the total number of diagnoses per patient, our analysis focused on the specific association of each diagnosis with mortality risk. This approach provides more granular insight, as each ND reflects distinct human responses and may be associated with distinct pathophysiological pathways. Identifying NDs associated with higher or lower mortality risk may inform care planning and prioritization in critical care settings. These findings suggest that standardized NDs could contribute to risk stratification frameworks in cardiac critical care.

We found that the ND of excess fluid volume was linked to a higher risk of in-hospital mortality. In patients with heart failure or ventricular dysfunction, fluid overload increases filling pressures and promotes pulmonary congestion and impaired oxygenation, thereby aggravating myocardial dysfunction and systemic compromise. Additionally, excess fluid reduces responsiveness to diuretics, impairs renal function, and increases the need for mechanical ventilation, further heightening the risk of complications [56,57].

Impaired cardiac output, reflecting the heart’s reduced capacity to maintain adequate tissue perfusion, was also associated with greater mortality. This dysfunction, common in acute myocardial infarction, results in systemic hypoperfusion and maladaptive compensatory responses that exacerbate myocardial stress and organ dysfunction [58].

Patients at risk of cardiovascular function deterioration and shock also had higher mortality. The former reflects the potential for oxygen supply–demand imbalance due to compromised cardiac performance, a frequent scenario in the CICU, leading to progressive hypoperfusion and neurohormonal activation [58]. The latter identifies patients at imminent risk of severe circulatory collapse, such as cardiogenic shock, characterized by profound tissue hypoxia, lactic acidosis, endothelial injury, and rapid organ dysfunction [59,60]. Both NDs denote unstable hemodynamic states and serve as clinically meaningful indicators of poor prognosis.

In this study, risk for decreased cardiac tissue perfusion was not significantly associated with in-hospital mortality. Although clinically plausible in severe cardiac conditions such as myocardial ischemia or arrhythmias [61,62], its association with mortality in this cohort was not evident. This diagnosis may represent a potential vulnerability rather than an established dysfunction and may have been documented preventively in the absence of overt clinical deterioration. Its similar prevalence among survivors and non-survivors further suggests limited ability to differentiate mortality risk in this setting.

Among extracardiac responses, risk for electrolyte imbalance was associated with higher mortality. Electrolyte disturbances (i.e., sodium, potassium, calcium, magnesium) are common in severe cardiac disease due to diuretic therapy, metabolic acidosis, or intravenous fluid administration. These alterations can precipitate arrhythmias, myocardial dysfunction, and hemodynamic instability, increasing the risk of fatal events [63].

We found that impaired gas exchange was also associated with higher mortality. Pulmonary congestion resulting from elevated capillary pressures compromises oxygenation and carbon dioxide elimination, leading to hypoxemia and acidosis. These disturbances can further impair myocardial contractility and precipitate arrhythmias, contributing to clinical deterioration [64,65].

We observed that the ND of risk for ineffective cerebral perfusion was associated with increased mortality. In cardiac patients, reduced cardiac output, arrhythmias, or cardiogenic shock may compromise cerebral blood flow, resulting in hypoxia, altered consciousness, and potentially irreversible neurological injury [66,67].

Patients diagnosed with anxiety, impaired psychological comfort, or acute pain showed a lower risk of in-hospital mortality. These findings should be interpreted with caution. Rather than suggesting a protective effect, these NDs likely reflect lower clinical severity at the time of assessment. Such NDs are typically documented in patients who are conscious, oriented, and hemodynamically stable enough to perceive and express emotional or physical discomfort. In contrast, critically ill patients with severe hemodynamic compromise, altered consciousness, or deep sedation are less likely to manifest or be assessed for these responses. Therefore, these NDs may function as indirect indicators of preserved physiological stability rather than independent determinants of improved survival [68].

Similarly, the inverse association observed between risk of unstable blood glucose and mortality should be interpreted with caution. Although glycemic instability is generally associated with adverse outcomes, this diagnosis may have been more readily documented in patients with preserved physiological status and active metabolic surveillance. Thus, the lower mortality observed in this group may reflect both less advanced clinical deterioration and intensified surveillance, rather than a true protective effect of the diagnosis itself [69,70].

The stratified analysis further supported the robustness of these associations and demonstrated that the association between NDs and in-hospital mortality was not uniform across levels of physiological severity. The statistically significant interaction terms between each ND and the shock index indicate true effect modification rather than random variation. Among patients with greater hemodynamic compromise (shock index ≥ 0.7), NDs reflecting direct or indirect cardiac dysfunction were associated with substantially higher relative risks, identifying a particularly vulnerable subgroup. Conversely, the inverse associations observed for emotional and comfort-related NDs were attenuated with increasing clinical severity, supporting the interpretation that these associations reflect preserved physiological stability rather than protective effects. Interestingly, acute pain showed a more pronounced inverse association among patients with higher shock index values, likely reflecting the preservation of consciousness and systemic perfusion required for pain perception. Overall, these findings suggest that NDs capture distinct, severity-dependent clinical dimensions associated with mortality, underscoring their potential as dynamic clinical indicators in critical care settings.

Beyond their clinical plausibility, these findings extend existing evidence. Although prior studies in chronic or cardiovascular populations have assessed mortality or length of stay using the total number of NDs as a single predictor, limited research has examined the specific association of individual diagnoses with mortality outcomes [23,27,28]. Only three prior studies (conducted in patients with COVID-19) have examined mortality by specific NDs, with mixed results [18,20,67]. By focusing on the individual effects of each diagnosis, our study extends this line of research to a cardiac critical care context and supports the use of standardized NDs as a structured framework for operationalizing gradients of physiological and psychosocial severity. This reinforces the potential of NDs to complement traditional severity indices in predictive modeling.

### 4.1. Limitations

For the proper interpretation of our findings, some limitations must be taken into consideration. First, due to its retrospective design, the observed associations cannot be interpreted as causal. However, clinical records were selected based on a diagnosis of heart disease rather than mortality, reducing the likelihood of selection bias.

Second, this was a single-center study conducted in a tertiary referral hospital. Although this institution manages a broad spectrum of acute cardiac conditions, its organizational structure, staffing models, documentation practices, and case mix may differ from those of other hospitals. Therefore, the transferability of these findings to other clinical settings, particularly those with different resource levels or nursing workforce compositions, should be interpreted with caution.

Third, as in all retrospective cohorts, data quality relied on the accuracy of documentation by healthcare personnel. In the Mexican healthcare context, nursing staff’s competence in using standardized language varies with training level. At the same time, registered nurses receive formal education in taxonomies such as NANDA-I, and nursing assistants typically do not. However, unlike in other healthcare systems, both groups often perform similar clinical tasks in practice and may share responsibility for patient care documentation, including NDs. As a result, critically ill patients were likely attended by both. Any errors in the identification or recording of diagnoses are likely non-differential and thus unlikely to introduce information bias.

Additionally, the applicability of NANDA-I NDs has been questioned in prior literature due to limitations, including limited empirical support, conceptual ambiguity, and poor adaptability to healthcare systems outside the U.S. [71,72,73,74,75]. These issues may affect its relevance in settings with different resources, priorities, or cultural models of care. Nonetheless, the taxonomy is continuously updated to improve clarity, validity, and applicability. Promoting rigorous research to provide stronger empirical support for NANDA-I is essential for justifying its use and adaptation across diverse clinical contexts.

Despite these limitations, the study’s methodological rigor strengthens confidence in the findings. The analytical approach incorporated a prespecified confounder structure grounded in DAGs, enhancing internal validity and reducing the risk of model misspecification. Importantly, we conducted an interaction analysis using the shock index (rather than adjusting for it as a confounder) to evaluate whether the association between each ND and in-hospital mortality varied by physiological severity. Adjusting for severity indicators, such as the shock index, would not have been appropriate, as these variables reflect the same underlying clinical instability captured by multiple NDs. Controlling them could therefore introduce collinearity and artificially attenuate true associations. Furthermore, the observed patterns are consistent with established pathophysiological mechanisms, supporting their clinical coherence and interpretability. Together, these elements strengthen the internal consistency of our findings and provide clinically relevant evidence on how individual NANDA-I NDs relate to mortality in cardiac critical care, offering a foundation for future prospective studies to further examine their potential clinical utility.

### 4.2. Implications for Nursing Practice

The findings of this study underscore the importance of clinical nursing judgment and the systematic application of standardized nursing language in CICUs. Beyond organizing care, NDs were consistently associated with in-hospital mortality and may provide complementary information for clinical risk assessment when interpreted alongside traditional severity indicators. Their integration allows a more comprehensive evaluation of patients by incorporating human responses that reflect functional, emotional, and hemodynamic states.

To ensure appropriate use, institutions should promote accurate and timely documentation of NDs, supported by continuous clinical observation and professional judgment. This requires investment in staff training, continuing education, supervision, and feedback mechanisms.

From an educational perspective, nursing curricula should strengthen clinical reasoning and critical thinking to support the rigorous and context-sensitive application of NANDA-I NDs. These diagnoses should not be viewed solely as academic frameworks, but as structured clinical assessments that may inform patient management in complex care settings.

Finally, further research is warranted to replicate these findings in multicenter, prospective studies. Future studies should examine additional outcomes such as length of hospital stay, readmissions, need for mechanical ventilation, and post-discharge quality of life. It is also important to evaluate whether incorporating NDs into structured risk assessment frameworks improves clinical decision-making. Moreover, the reliability and validity of NDs documented in routine practice require systematic evaluation, including interprofessional concordance and their temporal relationship with clinical events. Investigating barriers and facilitators to the appropriate implementation of the NCP in highly complex settings, such as CICUs, remains essential.

## 5. Conclusions

The results of this study suggest that NDs based on the NANDA-I taxonomy are independently associated with in-hospital mortality among critically ill patients with cardiac disease. Their systematic documentation may support early recognition of clinical risk and the identification of patients with greater physiological instability in intensive care settings. These findings highlight the central role of nursing clinical judgment in highly complex environments and suggest that NDs could complement traditional severity indicators within structured clinical assessment frameworks. However, given the limited evidence on the association between individual NDs and clinical outcomes, further high-quality prospective studies are needed to confirm and extend these findings.

## Figures and Tables

**Table 1:** Sociodemographic characteristics according to mortality status.

Characteristics	Total (n = 195)	Survivors n = 148 (75.9%)	Deaths n = 47 (24.1%)	*p*-Value ^a^
Sex, f (%)				
Women	83 (42.6)	63 (76.0)	20 (24.0)	0.999
Men	112 (57.4)	85 (75.9)	27 (24.1)	
Age (in years)				
Median (IQR)	53 (18)	61 (21)	67 (22)	0.014
Marital status, f (%)				
Unmarried	40 (20.5)	32 (80.0)	8 (20.0)	0.496
Married	155 (79.5)	116 (78.4)	39 (25.2)	
Educational level, f (%)				
No education	18 (9.2)	10 (55.6)	8 (44.4)	0.034
Basic education	63 (32.3)	44 (69.8)	19 (30.2)	
Intermediate level	73 (37.4)	62 (84.9)	11 (15.1)	
Higher education	41 (21.0)	32 (79.0)	9 (21.9)	
Employment status, f (%)				
Paid work	89 (45.6)	75 (83.2)	15 (16.8)	0.001
Retired	28 (14.4)	11 (39.3)	17 (60.7)	
Other	78 (40.0)	63 (80.8)	15 (19.2)	

Abbreviations: f, frequency; IQR, interquartile range. ^a^ Comparisons by in-hospital mortality status were performed using Pearson’s chi-squared test for categorical variables and the Mann–Whitney U test for median differences.

**Table 2:** Admission clinical characteristics stratified by mortality outcome.

Clinical Characteristics	Total (n = 195)	Survivors n = 148 (75.9%)	Deaths n = 47 (24.1%)	*p*-Value ^a^
Medical diagnosis, f (%)				
Ischemic heart disease	71 (36.4)	63 (88.7)	8 (11.3)	0.013
Rhythm and conduction disorders	29 (14.9)	20 (69.0)	9 (31.0)	
Valvular heart disease	38 (19.5)	24 (63.2)	14 (36.8)	
Heart failure and complications	42 (21.5)	29 (69.1)	13 (30.9)	
Postoperative cardiovascular status	15 (7.7)	12 (80.0)	3 (20.0)	
SBP (mmHg)				
Median (IQR)	124 (20)	124 (18)	120 (22)	0.004
DBP (mmHg)				
Median (IQR)	70 (11)	73 (5)	70 (11)	0.076
Body Temperature (degrees Celsius)				
Median (IQR)	36.5 (0.7)	36.5 (0.7)	36.5 (0.9)	0.168
Heart rate				
Median (IQR)	84 (14)	83 (13)	88(14)	0.013
Respiratory rate				
Median (IQR)	19 (3)	19 (2)	21 (20)	<0.001
Oxygen saturation				
Median (IQR)	95 (3)	95 (4)	93 (3)	0.002
Shock index				
≤0.7	129 (67.2)	114 (88.4)	15 (11.6)	<0.001
>0.7	66 (32.8)	34 (51.5)	32 (48.5)	
Comorbidities, f (%)				
None	64 (32.8)	57 (89.1)	7 (10.9)	0.011
One	94 (48.2)	65 (69.1)	29 (30.9)	
Two or more	37 (19.0)	26 (70.3)	11 (29.7)	
Length of Hospital Stay				
Median (IQR)	3 (2)	2 (2)	3 (1)	0.031

Abbreviations: SBP, systolic blood pressure; DBP, diastolic blood pressure; mmHg, millimeters of mercury; f, frequency; IQR, interquartile range. ^a^ Differences by in-hospital mortality were assessed using Pearson’s chi-squared test or Fisher’s exact test for categorical variables and the Mann–Whitney U test for the difference in medians.

**Table 3:** NANDA-I diagnostic labels by domains and class.

Domains	Class and Diagnostic Label (Code)	Total (n = 195)	
		f	%
DOMAIN 1. Health promotion	Class 2. Health management		
	Willingness to improve self-management of health (00293)	27	14.0
DOMAIN 2. Nutrition	Class 4. Metabolism		
	Risk for unstable blood glucose (00179)	52	26.7
	Class 5. Hydration		
	Risk for electrolyte imbalance (00195)	41	21.0
	Excess fluid volume (00026)	36	18.5
DOMAIN 3. Elimination and exchange	Class 1: Urinary function		
	Impaired urinary elimination (00016)	19	9.7
	Class 4. Respiratory function		
	Impaired gas exchange (00030)	61	31.3
DOMAIN 4. Activity/rest	Class 3. Energy balance		
	Decreased activity tolerance (00298)	35	18.0
	Class 4. Cardiovascular and pulmonary responses		
	Decreased cardiac output (00029)	111	56.9
	Risk for decreased cardiac tissue perfusion (00200)	158	81.0
	Risk for ineffective cerebral tissue perfusion (00201)	28	14.4
	Risk for impaired cardiovascular function (00311)	66	33.8
	Ineffective breathing pattern (00032)	55	28.1
DOMAIN 9. Coping/stress tolerance	Class 2. Coping responses		
	Anxiety (00146)	49	25.1
DOMAIN 11. Safety/protection	Class 1. Infection		
	Risk for infection (00004)	38	19.5
	Class 2. Physical injury		
	Risk for bleeding (00303)	25	12.8
	Risk for shock (00205)	57	29.2
	Class 6. Thermoregulation		
	Decreased body temperature (00472)	21	10.7
DOMAIN 12. Comfort	Class 1. Physical comfort		
	Acute pain (00132)	56	28.7
	Class 4. Psychological comfort		
	Impaired psychological comfort (00379)	41	21.0

Abbreviations: f, frequency.

## Data Availability

The original data presented in the study are openly available in [Mendeley Data] at [https://doi.org/10.17632/8chfgt8vwh.1].

## Supplementary Materials

The following supporting information can be downloaded at: https://www.mdpi.com/article/10.3390/clinpract16030049/s1, Figure S1. Directed acyclic graph (DAG) to explore the association between nursing diagnoses and in-hospital mortality in patients with cardiac disease; Figure S2. Crude risk ratio of the association between nursing diagnoses and in-hospital mortality in patients with cardiac disease.; Table S1. Nursing diagnoses according to mortality status; Table S2. Stratified analysis of the association between nursing diagnoses and mortality according to the Shock Index.

## Abbreviations

The following abbreviations are used in this manuscript:

ND	Nursing diagnoses
NCP	Nursing Care Process
CICU	Coronary intensive care units
CVDs	Cardiovascular diseases
RR	Relative Risk
CI	Confidence interval
DAG	Directed acyclic graphs
NANDA-I	North American Nursing Diagnosis Association-International
