# Supplementary material for: Association Between Nursing Diagnoses and Mortality in Patients with Cardiac Disease: A Retrospective Cohort Study
Source: Clin Pract. 2026 Feb 26;16(3):49. doi: 10.3390/clinpract16030049 (PMC13025170; doi:10.3390/clinpract16030049)
Supplement: Supplementary file 1 [file clinpract-16-00049-s001.zip › Figure S1.pdf]

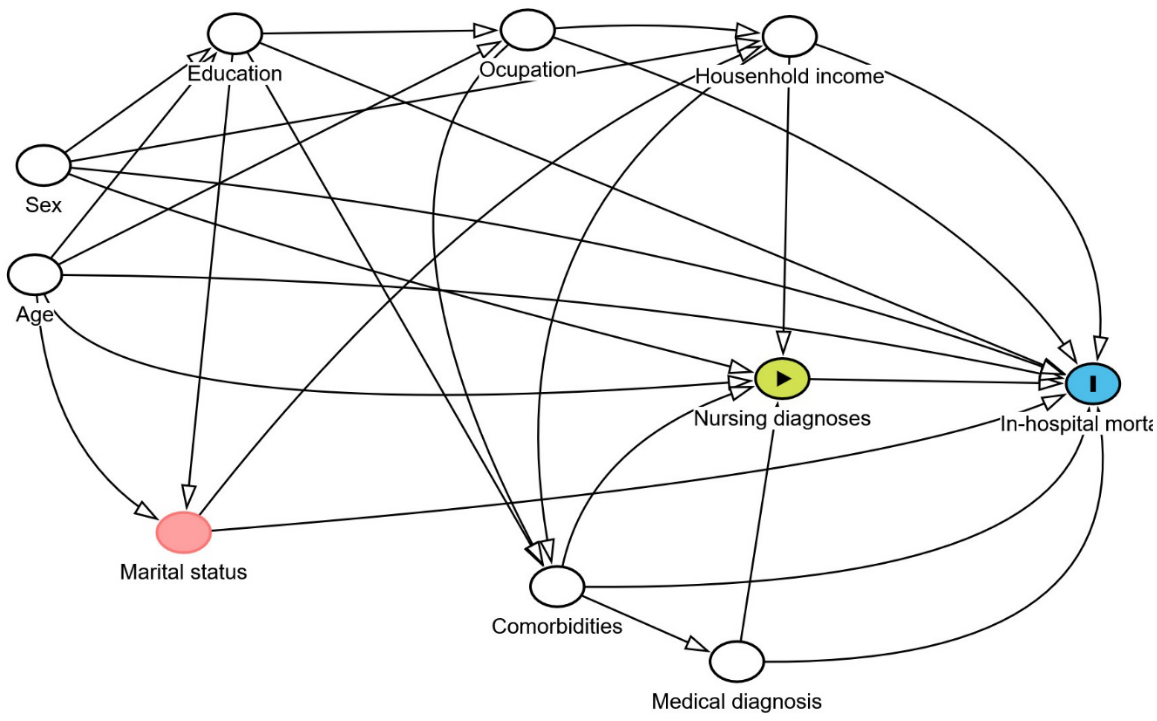

Figure S1. DAG to explore the association between nursing diagnoses and in-hospital mortality in patients with cardiac disease.
