# Supplementary material for: Association Between Nursing Diagnoses and Mortality in Patients with Cardiac Disease: A Retrospective Cohort Study
Source: Clin Pract. 2026 Feb 26;16(3):49. doi: 10.3390/clinpract16030049 (PMC13025170; doi:10.3390/clinpract16030049)
Supplement: Supplementary file 1 [file clinpract-16-00049-s001.zip › Table S2.pdf]

Table S2. Stratified analysis of the association between nursing diagnoses and mortality according to the Shock Index

Nursing Diagnosis	Shock Index < 0.7		Shock Index ≥ 0.7		P for interaction
	Adjusted RR (95% CI)	p-value	Adjusted RR (95% CI)	p-value	
**Risk for unstable blood glucose**	0.15 (0.02, 1.10)	0.060	0.60 (0.19, 1.88)	0.388	0.001
**Risk for electrolyte imbalance**	2.32 (1.21, 4.45)	0.010	2.86 (1.39, 6.53)	0.005	0.004
**Excess fluid volume**	1.87 (0.77, 4.40)	0.169	2.88 (0.86, 9.59)	0.087	<0.001
**Impaired gas exchange**	2.04 (1.06, 3.92)	0.031	2.86 (1.48, 6.26)	0.002	<0.001
**Decreased cardiac output**	1.67 (1.21, 2.29)	0.002	1.85 (1.25, 2.70)	0.021	0.005
**Risk for ineffective cerebral tissue perfusion**	1.32 (0.76, 1.49)	0.283	7.31 (3.02, 17.68)	0.001	<0.001
**Risk for impaired cardiovascular function**	1.47 (0.84, 2.58)	0.171	2.35 (1.32, 4.17)	0.004	0.003
**Anxiety**	0.21 (0.03, 1.40)	0.109	0.58 (0.30, 1.42)	0.117	0.001
**Risk for shock**	3.09 (1.75, 5.49)	0.001	4.55 (1.77, 11.72)	0.002	<0.001
**Acute pain**	0.62 (0.25, 1.22)	0.297	0.16 (0.03, 0.79)	0.024	0.002
**Impaired psychological comfort**	0.29 (0.12, 0.67)	0.004	0.80 (0.10, 1.02)	0.052	0.001

Abbreviations: RR, risk ratio; CI, confidence interval.
