# Supplementary material for: Association Between Nursing Diagnoses and Mortality in Patients with Cardiac Disease: A Retrospective Cohort Study
Source: Clin Pract. 2026 Feb 26;16(3):49. doi: 10.3390/clinpract16030049 (PMC13025170; doi:10.3390/clinpract16030049)
Supplement: Supplementary file 1 [file clinpract-16-00049-s001.zip › Table S1.pdf]

Table S1. Nursing diagnoses according to mortality status

Nursing Diagnoses	In-hospital mortality			
	Total (n=195)	Survivors n=148 (75.9%)	Deaths n=47 (24.1%)	*P-value*[a]
**Willingness to improve self-management of health**				
Absent	168 (86.2)	126 (75.0)	42 (25.0)	0.070
Present	127 (13.8)	22 (81.5)	5 (18.5)	
**Risk for unstable blood glucose**				
Absent	143 (73.3)	101 (70.6)	42 (29.4)	0.004
Present	52 (26.7)	47 (90.4)	5 (9.6)	
**Risk for Electrolyte Imbalance**				
Absent	154 (79.0)	125 (81.2)	29 (18.8)	0.001
Present	41 (21.0)	23 (56.1)	18 (43.9)	
**Excess fluid volume**				
Absent	159 (81.5)	125 (78.6)	34 (21.4)	0.062
Present	36 (18.5)	23 (63.9)	13 (36.1)	
**Impaired urinary elimination**				
Absent	176 (90.3)	135 (76.7)	41 (23.3)	0.423
Present	19 (9.7)	13 (68.4)	6 (31.6)	
**Impaired gas exchange**				
Absent	134 (68.7)	112 (83.6)	22 (16.4)	0.001
Present	61 (31.3)	36 (59.0)	25 (41.0)	
**Decreased activity tolerance**				
Absent	160 (82.1)	126 (78.8)	34(21.2)	0.050
Present	35 (17.9)	22 (62.9)	13 (37.1)	
**Decreased cardiac output**				
Absent	84 (43.1)	78 (92.9)	6 (7.1)	
Present	111 (56.9)	70 (63.1)	41 (36.9)	0.001
**Risk for decreased cardiac tissue perfusion**				
Absent	37 (19.0)	28 (75.7)	9 (24.3)	0.972
Present	158 (81.1)	120 (75.9)	38 (24.1)	
**Risk for ineffective cerebral tissue perfusion**				
Absent	168 (86.2)	126 (79.6)	34 (20.4)	0.003
Present	127 (13.8)	15 (53.6)	13 (46.4)	
**Risk for impaired cardiovascular function**				
Absent	168 (86.2)	107 (82.9)	22 (17.1)	0.001
Present	127 (13.8)	41 (62.1)	25 (37.9)	
**Ineffective breathing pattern**				
Absent	140 (71.8)	106 (75.7)	34 (24.3)	0.924
Present	55 (28.2)	42 (76.4)	13 (23.7)	
**Anxiety**				
Absent	144 (73.8)	104 (72.2)	40 (27.8)	
Present	51 (26.2)	44 (86.3)	7 (13.7)	0.044
**Risk for infection**				

Absent	157 (80.5)	118 (75.2)	39 (24.8)	0.624
Present	38 (19.5)	30 (78.9)	8 (21.1)	
**Risk for bleeding**				
Absent	170 (87.2)	130 (76.5)	40 (23.5)	0.626
Present	25 (12.8)	18 (72.0)	7 (28.0)	
**Risk for shock**				
Absent	138 (70.7)	116 (84.1)	22 (15.9)	0.001
Present	57 (29.3)	32 (56.1)	25 (43.9)	
**Decreased body temperature**				
Absent	174 (89.2)	137 (78.7)	37 (21.3)	0.008
Present	21 (10.7)	11 (52.4)	10 (47.6)	
**Acute pain**				
Absent	168 (86.2)	97 (69.8)	42 (30.2)	0.002
Present	127 (13.8)	51 (91.1)	5 (8.9)	
**Impaired psychological comfort**				
Absent	154 (79.0)	110 (71.4)	44 (28.6)	0.005
Present	141 (21.0)	38 (92.7)	3 (7.3)	

[a] Comparing subjects by in-hospital mortality status using Pearson's chi-squared test or Fisher's exact test for categorical variables
