# Supplementary material for: Association Between Nursing Diagnoses and Mortality in Patients with Cardiac Disease: A Retrospective Cohort Study
Source: Clin Pract. 2026 Feb 26;16(3):49. doi: 10.3390/clinpract16030049 (PMC13025170; doi:10.3390/clinpract16030049)
Supplement: Supplementary file 1 [file clinpract-16-00049-s001.zip › Figure S2.pdf]

Figure S2. Crude risk ratio of the association between nursing diagnoses and in-hospital mortality in patients with cardiac disease.

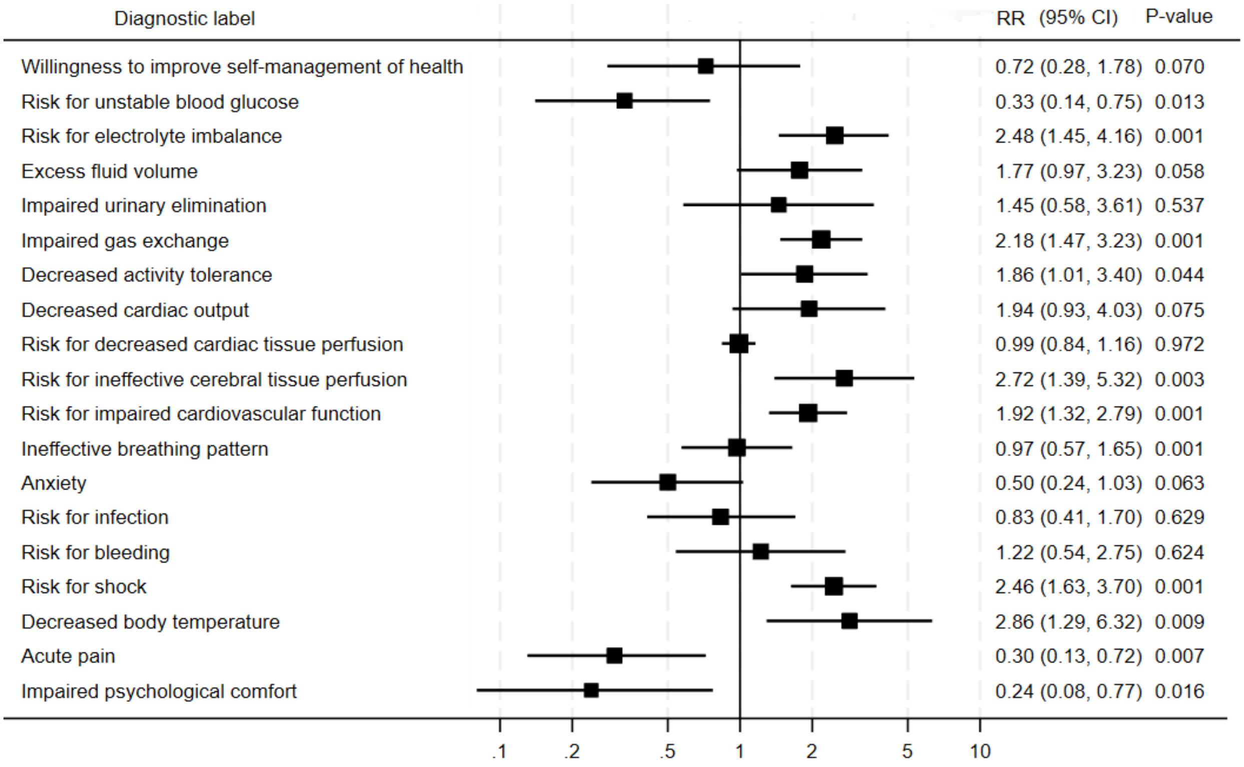

Footnote: Abbreviations: RR, risk ratio; CI, confidence interval.
The x-axis displays risk ratios ranging from 0.1 to 10, with RR = 1 indicating no association. Values <1 indicate an inverse association with in-hospital mortality, whereas values >1 indicate a positive association
